# Phylogenetic Analysis of the Spider Mite Sub-Family Tetranychinae (Acari: Tetranychidae) Based on the Mitochondrial COI Gene and the 18S and the 5′ End of the 28S rRNA Genes Indicates That Several Genera Are Polyphyletic

**DOI:** 10.1371/journal.pone.0108672

**Published:** 2014-10-07

**Authors:** Tomoko Matsuda, Maiko Morishita, Norihide Hinomoto, Tetsuo Gotoh

**Affiliations:** 1 Laboratory of Applied Entomology and Zoology, Faculty of Agriculture, Ibaraki University, Ibaraki, Japan; 2 NARO Agricultural Research Center, National Agriculture and Food Research Organization, Ibaraki, Japan; Tel-Aviv University, Israel

## Abstract

The spider mite sub-family Tetranychinae includes many agricultural pests. The internal transcribed spacer (ITS) region of nuclear ribosomal RNA genes and the cytochrome *c* oxidase subunit I (COI) gene of mitochondrial DNA have been used for species identification and phylogenetic reconstruction within the sub-family Tetranychinae, although they have not always been successful. The 18S and 28S rRNA genes should be more suitable for resolving higher levels of phylogeny, such as tribes or genera of Tetranychinae because these genes evolve more slowly and are made up of conserved regions and divergent domains. Therefore, we used both the 18S (1,825–1,901 bp) and 28S (the 5′ end of 646–743 bp) rRNA genes to infer phylogenetic relationships within the sub-family Tetranychinae with a focus on the tribe Tetranychini. Then, we compared the phylogenetic tree of the 18S and 28S genes with that of the mitochondrial COI gene (618 bp). As observed in previous studies, our phylogeny based on the COI gene was not resolved because of the low bootstrap values for most nodes of the tree. On the other hand, our phylogenetic tree of the 18S and 28S genes revealed several well-supported clades within the sub-family Tetranychinae. The 18S and 28S phylogenetic trees suggest that the tribes Bryobiini, Petrobiini and Eurytetranychini are monophyletic and that the tribe Tetranychini is polyphyletic. At the genus level, six genera for which more than two species were sampled appear to be monophyletic, while four genera (*Oligonychus*, *Tetranychus*, *Schizotetranychus* and *Eotetranychus*) appear to be polyphyletic. The topology presented here does not fully agree with the current morphology-based taxonomy, so that the diagnostic morphological characters of Tetranychinae need to be reconsidered.

## Introduction

The spider mite sub-family Tetranychinae includes some pests that cause serious economic losses throughout the world [1], [2], [3]. The family consists of more than 1,200 species, some of which have a wide host range, whereas others are highly host-specific [4], [5]. For example, *Tetranychus urticae* Koch, *Panonychus citri* (McGregor) and *Oligonychus coffeae* (Nietner), have an especially strong effect on agricultural and horticultural crops, and they are polyphagous. However, these genera also include mono-, oligophagous species, such as *Tetranychus bambusae* Wang & Ma, *Panonychus bambusicola* Ehara & Gotoh, *Oligonychus orthius* Rimando, *Oligonychus modestus* (Banks) and *Oligonychus rubicundus* Ehara which inhabit only gramineous plants.

Although exact species identification is the first step in any biological study, spider mites are difficult to distinguish by morphological characters alone because of their small size (<0.5 mm) and limited number of diagnostic characters [6], [7], [8]. Therefore, the use of DNA-based methods to identify species has increasingly been used for some genera of the Tetranychinae. For example, Navajas and Boursot [9] showed that *T*. *urticae* and *Tetranychus turkestani* Ugarov & Nikolskii, which are very closely related species, can be identified by using the internal transcribed spacer 2 (ITS2) region of nuclear ribosomal RNA (rRNA) genes. More recently, Matsuda et al. [10], [11] revealed that almost all species of Japanese *Oligonychus* (17 of 18 species) and all species of *Tetranychus* (13 species) can be identified by using the cytochrome *c* oxidase subunit I (COI) gene of mitochondrial DNA.

Despite recent advances in DNA-based methods for identifying spider mites, most phylogenetic relationships of sub-families, tribes and genera of the Tetranychinae remain poorly understood, as is reflected by the low support values for most nodes of the phylogenetic trees. However, phylogenetic trees clearly show that the genus *Oligonychus* is polyphyletic. Navajas et al. [12] and Ros and Breeuwer [13] analyzed the phylogeny of Tetranychinae including three *Oligonychus* species (*Oligonychus ununguis* (Jacobi), *Oligonychus platani* (McGregor) and *Oligonychus gossypii* (Zacher)) using the COI gene. Although these three species have the same empodium shape, *O. gossypii*, whose aedeagus curves dorsally, can be easily distinguished from *O*. *ununguis* and *O*. *platani* whose aedeagi curve ventrally. In the phylogenetic trees of these two studies, *O*. *gossypii* clustered more closely with *Tetranychus* species whose aedeagi also curve dorsally, while *O*. *ununguis* and *O*. *platani* formed a separate group. Polyphyly in the genus *Oligonychus* was also reported in the ITS2 region [14].

The unresolved phylogeny among the taxa of the sub-family Tetranychinae based on the COI sequences is probably due to the strongly biased nucleotide composition and the saturation at the third codon positions [13]. Because both the 18S and 28S rRNA genes evolve more slowly and are made up of conserved regions and divergent domains [15], these genes have been used for phylogenetic analyses of higher taxonomic relationships (from “phyla” to “classes” within Ecdysozoa) [16], [17]. In resolving tick genera (Acari: Ixodida), combining the 18S and 28S rRNA genes provided more detailed relationships than did the 18S gene alone [18], [19]. Therefore, we used both the 18S (1,825–1,901 bp) and 28S (the 5′ end of 646–743 bp) rRNA genes to infer phylogenetic relationships within the sub-family Tetranychinae. Then, we compared the trees based on the 18S and 28S genes with the tree based on the mitochondrial COI gene (618 bp). Another problem in previous studies [12], [13], [14] was that only 16 to 25 species were used for the phylogenetic analyses. Limited taxon sampling can seriously influence the resulting phylogenetic inferences (for reviews, see [20], [21], [22]). Therefore, to assess the phylogenetic relationships among tribes and genera of the sub-family Tetranychinae, we examined a total of 88 strains (15 genera and 4 tribes) most of which were from Japan.

## Results

### Mitochondrial COI gene

**Figure 1:**
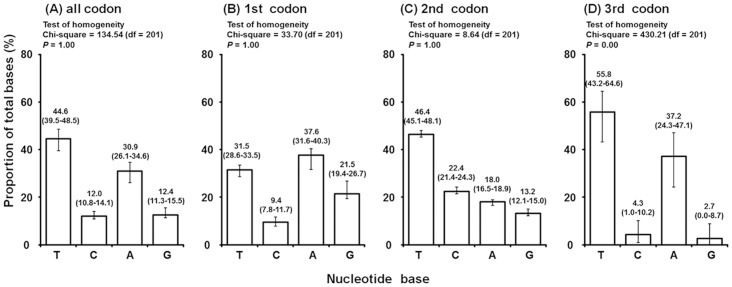
Base compositions of the codons of the mitochondrial COI gene. (A) All codon positions, (B) 1st codon position, (C) 2nd codon position, (D) 3rd codon position, averaged over all 68 mite strains used in this study. Error bars depict range. Results of the homogeneity test are given for each codon position.

We obtained the COI sequences of 38 strains determined in this study (Table 1) and 30 strains from previously published data [10], [11]. The COI sequences contained no insertions or deletions. After alignment, the COI fragment had 618 nucleotides, of which 282 were parsimony-informative sites (File S1). The AT contents of the COI sequences of the tetranychid mites were very high (75.5%), especially at the 3rd codon position (93.0%). Chi-square tests revealed no significant heterogeneity in the first and second codon positions of the COI sequences, but significant heterogeneity at third codon positions (Figure 1). Similar high AT contents have been observed in previous studies of tetranychid mites [10], [11], [12], [13].

**Table 1:** Classification and sources of Tetranychid mites used in this study.

Sub-family	Tribe	Genus	Species	Date	Locality	Host plant	Voucher specimen no.^a^	Accession no.		
								COI	18S	28S
Bryobiinae	Bryobiini	*Bryobia*	*B*. *eharai* Pritchard & Keifer	Sept. 11, 2012	Ibaraki, Japan	*Chrysanthemum morifolium*	0612	–	AB926227	AB926318
			*B. praetiosa* Koch	July 27, 2008	Hokkaido, Japan	*Trifolium repens*	0609	AB981203	AB926228	AB926319
	Petrobiini	*Petrobia*	*P*. *latens* (Müller)	Mar. 30, 2012	Tokushima, Japan	*Daucus carota*	0482	AB981204	AB926229	AB926320
		*Tetranychina*	*T*. *harti* (Ewing)	June 11, 2012	Ibaraki, Japan	*Oxalis corniculata*	0602	–	AB926230	AB926321
Tetranychinae	Eurytetranychini	*Eurytetranychoides*	*E*. *japonicus* (Ehara)	Sept. 22, 2010	Tokyo, Japan	*Lithocarpus edulis*	0493	AB981205	AB926231	AB926322
		*Eutetranychus*	*E*. *africanus* (Tucker)	June 30, 2008	Taichung, Taiwan	*Pueraria montana*	0377	–	AB926232	AB926323
		*Aponychus*	*A*. *corpuzae* Rimando	Apr. 10, 2001	Ibaraki, Japan	*Sasa senanensis*	0607	AB981206	AB926233	AB926324
			*A*. *firmianae* (Ma & Yuan)	Aug. 7, 2010	Ibaraki, Japan	*Firmiana simplex*	0405	–	AB926234	AB926325
	Tetranychini	*Panonychus*	*P*. *bambusicola* Ehara & Gotoh	June 4, 1989	Hokkaido, Japan	*Sasa senanensis*	0606	AB981207	AB926235	AB926326
			*P*. *caglei* Mellot	Aug. 19, 2009	Okinawa, Japan	*Trichosanthes pilosa*	0611	–	AB926236	AB926327
			*P*. *citri* (McGregor)	May 6, 1993	Ibaraki, Japan	*Ilex crenata*	0226	AB981208	AB926237	AB926328
			*P*. *elongatus* Manson	July 27, 2010	Hangzhou, China	*Broussonetia papyrifera*	0398	–	AB926238	AB926329
			*P*. *mori* Yokoyama	Apr. 22, 2007	Hokkaido, Japan	*Morus australis*	0239	AB981209	AB926239	AB926330
			*P*. *osmanthi* Ehara & Gotoh	Nov. 16, 2001	Guilin, China	*Osmanthus fragrans*	0229	AB981210	AB926240	AB926331
			*P*. *thelytokus* Ehara & Gotoh	Aug. 4, 2010	Hokkaido, Japan	*Ulmus davidiana*	0407	AB981211	AB926241	AB926332
			*P*. *ulmi* (Koch)	Aug. 2, 2012	Nagano, Japan	*Malus pumila*	0603	AB981212	AB926242	AB926333
		*Sasanychus*	*S*. *akitanus* (Ehara)	June 23, 1986	Hokkaido, Japan	*Sasa senanensis*	0605	AB981213	AB926243	AB926334
			*S*. *pusillus* Ehara & Gotoh	July 31, 2012	Hokkaido, Japan	*Sasa chartacea*	0575	AB981214	AB926244	AB926335
		*Schizotetranychus*	*S*. *bambusae* Reck	Aug. 27, 2011	Chiba, Japan	*Phyllostachys edulis*	0503	AB981215	AB926245	AB926336
			*S*. *brevisetosus* Ehara	Oct. 13, 2011	Kochi, Japan	*Quercus glauca*	0527	AB981216	AB926246	AB926337
			*S*. *cercidiphylli* Ehara	Aug. 3, 2010	Hokkaido, Japan	*Cercidiphyllum japonicum*	0411	AB981217	AB926247	AB926338
			*S*. *gilvus* Ehara & Ohashi	May 22, 2012	Nara, Japan	*Quercus gilva*	0549	AB981218	AB926248	AB926339
			*S*. *lespedezae* Begljarov & Mitrofanov	Aug. 26, 2011	Ibaraki, Japan	*Pueraria montana*	0515	AB981219	AB926249	AB926340
			*S*. *recki* Ehara	Aug. 4, 2010	Hokkaido, Japan	*Sasa senanensis*	0408	AB981220	AB926250	AB926341
			*S*. *schizopus* (Zacher)	June 14, 2010	Tokyo, Japan	*Salix integra*	0532	AB981221	AB926251	AB926342
			*S*. *shii* (Ehara)	June 14, 2010	Tokyo, Japan	*Castanopsis sieboldii*	0533	AB981222	AB926252	AB926343
		*Stigmaeopsis*	*S*. *celarius* Banks	Aug. 7, 2011	Ibaraki, Japan	*Pleioblastus chino*	0506	AB981223	AB926253	AB926344
			*S*. *longus* (Saito)	June 4, 1989	Hokkaido, Japan	*Sasa senanensis*	0542	AB981224	AB926254	AB926345
			*S*. *miscanthi* (Saito)	Feb. 16, 2009	Nagasaki, Japan	*Miscanthus sinensis*	0404	AB981225	AB926255	AB926346
			*S*. *saharai* Saito & Mori	Aug. 5, 2011	Chiba, Japan	*Pleioblastus chino*	0501	AB981226	AB926256	AB926347
			*S*. *takahashii* Saito & Mori	Oct. 27, 1997	Hokkaido, Japan	*Sasa senanensis*	0541	AB981227	AB926257	AB926348
		*Yezonychus*	*Y*. *sapporensis* Ehara	Aug. 4, 2010	Hokkaido, Japan	*Sasa senanensis*	0406	AB981228	AB926258	AB926349
		*Eotetranychus*	*E*. *asiaticus* Ehara	Mar. 19, 2007	Nagasaki, Japan	*Citrus reticulata*	0546	AB981229	AB926259	AB926350
			*E*. *boreus* Ehara	June 3, 2010	Wakayama, Japan	*Armeniaca mume*	0415	–	AB926260	AB926351
			*E*. *celtis* Ehara	Aug. 27, 2011	Chiba, Japan	*Aphananthe aspera*	0502	AB981230	AB926261	AB926352
			*E*. *cornicola* Ehara	Aug. 5, 2011	Chiba, Japan	*Cornus controversa*	0498	AB981231	AB926262	AB926353
			*E*. *dissectus* Ehara	Aug. 3, 2010	Hokkaido, Japan	*Acer pictum*	0412	AB981232	AB926263	AB926354
			*E*. *nomurai* Ehara	Aug. 20, 2011	Ibaraki, Japan	*Celtis sinensis*	0514	AB981233	AB926264	AB926355
			*E*. *pruni* (Oudemans)	Sept. 1, 2012	Ibaraki, Japan	*Castanea crenata*	0562	–	AB926265	AB926356
			*E*. *querci* Reeves	Aug. 3, 2010	Hokkaido, Japan	*Tilia japonica*	0403	–	AB926266	AB926357
			*E*. *quercifoliae* Ehara & Gotoh	July 6, 2011	Ibaraki, Japan	*Quercus serrata*	0507	AB981234	AB926267	AB926358
			*E*. *rubricans* Ehara	Sept. 1, 2012	Ibaraki, Japan	*Carpinus tschonoskii*	0559	–	AB926268	AB926359
			*E*. *smithi* Pritchard & Baker	Aug. 14, 2007	Nagasaki, Japan	*Rosa multiflora*	0545	AB981235	AB926269	AB926360
			*E*. *spectabilis* Ehara	Sept. 7, 2011	Hokkaido, Japan	*Acer pictum*	0524	–	AB926270	AB926361
			*E*. *suginamensis* (Yokoyama)	Aug. 26, 2011	Ibaraki, Japan	*Morus australis*	0517	AB981236	AB926271	AB926362
			*E*. *tiliarium* (Hermann)	Aug. 3, 2010	Hokkaido, Japan	*Alnus hirsuta*	0409	–	AB926272	AB926363
			*E*. *toyoshimai* Ehara & Gotoh	Aug. 29, 2011	Iwate, Japan	*Magnolia obovata*	0519	–	AB926273	AB926364
			*E*. *uchidai* Ehara	Aug. 15, 2011	Hokkaido, Japan	*Ulmus davidiana*	0528	AB981237	AB926274	AB926365
			*E*. *uncatus* Garman	Aug. 3, 2010	Hokkaido, Japan	*Betula platyphylla*	0413	–	AB926275	AB926366
		*Oligonychus*	*O*. *amiensis* Ehara & Gotoh	July 13, 2005	Ibaraki, Japan	*Lithocarpus edulis*	0116	AB683672	AB926276	AB926367
			*O*. *biharensis* (Hirst)	Dec. 21, 2007	Okinawa, Japan	*Mangifera indica*	0012	AB683678	AB926277	AB926368
			*O*. *camelliae* Ehara & Gotoh	May 13, 2000	Fukushima, Japan	*Camellia japonica*	0082	AB683662	AB926278	AB926369
			*O*. *castaneae* Ehara & Gotoh	May 5, 2009	Ibaraki, Japan	*Castanea crenata*	0297	AB683667	AB926279	AB926370
			*O*. *clavatus* (Ehara)	July 28, 2009	Kanagawa, Japan	*Pinus thunbergii*	0360	AB683654	AB926280	AB926371
			*O*. *coffeae* (Nietner)	May 30, 2005	Okinawa, Japan	*Mangifera indica*	0078	AB683670	AB926281	AB926372
			*O*. *gotohi* Ehara	July 1, 2007	Ibaraki, Japan	*Lithocarpus edulis*	0076	AB683668	AB926282	AB926373
			*O*. *hondoensis* (Ehara)	Aug. 22, 2009	Aomori, Japan	*Cryptomeria japonica*	0376	AB683658	AB926283	AB926374
			*O*. *ilicis* (McGregor)	Oct. 30, 2000	Kagoshima, Japan	*Camellia sinensis*	0081	AB683660	AB926284	AB926375
			*O*. *karamatus* (Ehara)	Aug. 27, 2009	Hokkaido, Japan	*Larix kaempferi*	0358	AB683656	AB926285	AB926376
			*O*. *modestus* (Banks)	Sept. 9, 2008	Okinawa, Japan	*Digitaria ciliaris*	0092	AB683677	AB926286	AB926377
			*O*. *orthius* Rimando	July 9, 2009	Okinawa, Japan	*Saccharum officinarum*	0378	AB683675	AB926287	AB926378
			*O*. *perditus* Pritchard & Baker	Sept. 17, 2009	Kanagawa, Japan	*Juniperus* sp.	0364	AB683665	AB926288	AB926379
			*O*. *pustulosus* Ehara	Aug. 22, 2009	Aomori, Japan	*Cryptomeria japonica*	0363	AB683655	AB926289	AB926380
			*O*. *rubicundus* Ehara	Oct. 17, 2008	Kochi, Japan	*Miscanthus sinensis*	0290	AB683681	AB926290	AB926381
			*O*. *ununguis* (Jacobi)	July 27, 2008	Hokkaido, Japan	*Cryptomeria japonica*	0088	AB683664	AB926291	AB926382
		*Amphitetranychus*	*A*. *quercivorus* (Ehara & Gotoh)	July 9, 2003	Ibaraki, Japan	*Quercus crispula*	0610	AB981238	AB926292	AB926383
			*A*. *viennensis* (Zacher)	Sept. 21, 2010	Tokyo, Japan	*Armeniaca vulgaris*	0613	AB981239	AB926293	AB926384
		*Tetranychus*	*T*. *bambusae* Wang & Ma	July 5, 2009	Okinawa, Japan	*Phyllostachys edulis*	0343		AB926294	AB926385
			*T*. *evansi* Baker & Pritchard	Nov. 3, 2006	Tokyo, Japan	*Solanum nigrum*	0210	AB736039	AB926295	AB926386
			*T*. *ezoensis* Ehara	Sept. 3, 2008	Ibaraki, Japan	*Taxus cuspidata*	0281	AB736042	AB926296	AB926387
			*T*. *huhhotensis* Ehara, Gotoh & Hong	July 26, 2007	Inner Mongolia Autonomous Region, Mongolia	*Zea mays*	0201	–	AB926297	AB926388
			*T*. *kanzawai* Kishida	May 19, 1993	Shizuoka, Japan	*Thea sinensis*	0158	AB736043	AB926298	AB926389
			*T*. *lombardinii* Baker & Pritchard	July 10, 2008	Durban, South Africa	*Erythrina variegata*	0381	–	AB926299	AB926390
			*T*. *ludeni* Zacher	Oct.17, 1995	Ibaraki, Japan	*Solidago virgaurea*	0189	AB736051	AB926300	AB926391
			*T*. *macfarlanei* Baker & Pritchard	Sept. 30, 2008	Mymensingh, Bangladesh	*Dolichos lablab*	0389	–	AB926301	AB926392
			*T*. *merganser* Boudreaux	Apr. 6, 2007	El Talo, Sonora, Mexico	*Cucurbita maxima*	0225	–	AB926302	AB926393
			*T*. *misumaiensis* Ehara & Gotoh	Aug. 23, 2005	Hokkaido, Japan	*Apios* sp.	0218	AB736054	AB926303	AB926394
			*T*. *neocaledonicus* Andre	May 27, 1998	Tokyo, Japan	*Morus australis*	0192	AB736055	AB926304	AB926395
			*T*. *okinawanus* Ehara	June 19, 2003	Okinawa, Japan	*Pueraria montana*	0208	AB736058	AB926305	AB926396
			*T*. *parakanzawai* Ehara	June 5, 1993	Ibaraki, Japan	*Pueraria montana*	0155	AB736060	AB926306	AB926397
			*T*. *phaselus* Ehara	June 29, 2000	Ibaraki, Japan	*Glycine max*	0191	AB736066	AB926307	AB926398
			*T*. *piercei* McGregor	Dec. 20, 2007	Okinawa, Japan	*Cucumis melo*	0014	AB736068	AB926308	AB926399
			*T*. *pueraricola* Ehara & Gotoh	Oct. 23, 1993	Ibaraki, Japan	*Pueraria montana*	0203	AB736071	AB926309	AB926400
			*T*. *truncatus* Ehara	May 8, 2004	Kyoto, Japan	*Solanum nigrum*	0195	AB736075	AB926310	AB926401
			*T*. *turkestani* Ugarov & Nikolski	Sept. 15, 2007	Hamedan, Iran	*Phaseolus vulgaris*	0219	AB981240	AB926311	AB926402
			*T*. *urticae* Koch (green form)	July 16, 2001	Hokkaido, Japan	*Citrullus lanatus*	0181	AB736076	AB926312	AB926403
			*T*. *urticae* Koch (red form)	Aug. 27, 2001	Nagano, Japan	*Dianthus* sp.	0171	AB736079	AB926313	AB926404
			*T*. *zeae* Ehara, Gotoh & Hong	July 26, 2007	Inner Mongolia Autonomous Region, Mongolia	*Zea mays*	0202	–	AB926314	AB926405

*^a^*Voucher specimens are preserved at the Laboratory of Applied Entomology and Zoology, Faculty of Agriculture, Ibaraki University under the serial voucher specimen number.

**Figure 2:**
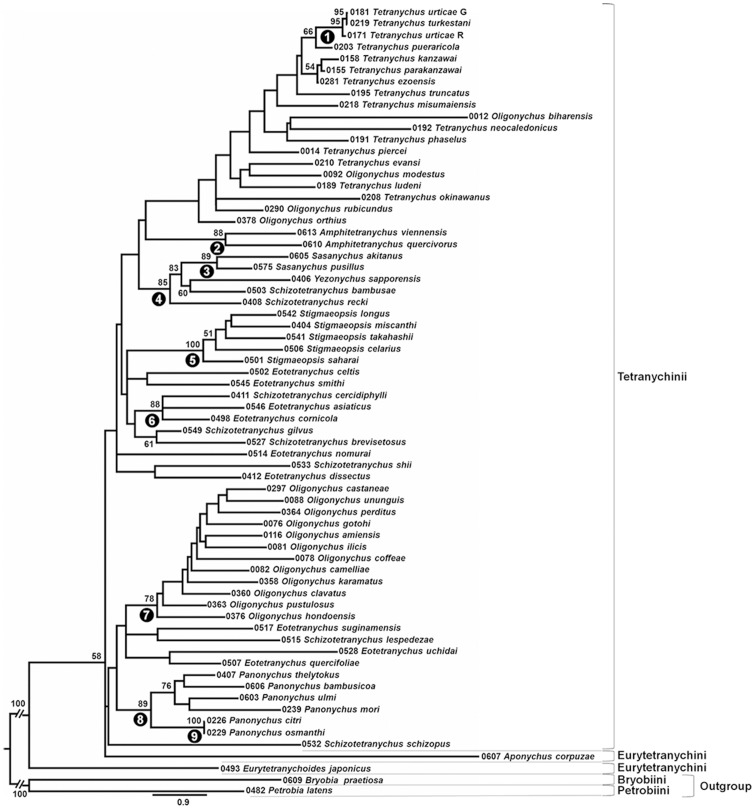
Maximum likelihood (ML) phylogenetic tree of the sub-family Tetranychinae based on the mitochondrial COI gene using the GTR Gamma model. Bootstrap values (>50%) based on 1,000 replications are indicated at nodes. Each operational taxonomic unit is indicated by the voucher specimen no. and scientific name. Black circles with numbers indicate the clade no. which corresponds with the article.

A phylogenetic tree of the sub-family Tetranychinae based on the COI gene is shown in Figure 2. Among the eight genera for which more than two strains were sampled, four genera (*Panonychus*, *Sasanychus*, *Stigmaeopsis* and *Amphitetranychus*) appear to be monophyletic with >80 bootstrap values, while the other four (*Oligonychus*, *Tetranychus*, *Schizotetranychus* and *Eotetranychus*) are polyphyletic. The four monophyletic genera are in clades 8, 3, 5 and 2, respectively (Figure 2). As was observed in previous studies, *Oligonychus* species whose aedeagus curves ventrally (clade 7) can be easily distinguished from *Oligonychus biharensis* (Hirst), *O*. *modestus*, *O*. *orthius* and *O*. *rubicundus* whose aedeagi curve dorsally. Although *Schizotetranychus* and *Eotetranychus* are scattered across the tree, some species formed well-supported clades. *Schizotetranychus bambusae* Reck & *Schizotetranychus recki* Ehara clustered with *Sasanychus* and *Yezonychus* species (clade 4). The clade including *Schizotetranychus cercidiphylli* Ehara, *Eotetranychus asiaticus* Ehara and *Eotetranychus cornicola* Ehara are supported with high bootstrap value (clade 6: bootstrap value (BP) = 88). The COI tree also shows monophyly of closely related species that morphologically and molecularly resemble each other, such as *P*. *citri* and *Panonychus osmanthi* Ehara & Gotoh [23], [24] (clade 9) and *T*. *urticae* and *T*. *turkestani* [9] (clade 1). These results are consistent with the 18S and 28S topologies described below. However, the COI phylogeny was not resolved and the deep-level relationships were especially unresolved, as shown by the low bootstrap values (Figure 2), as was observed in previous studies [12], [13]. The deep-level phylogeny of the sub-family Tetranychinae was also not resolved in the Bayesian tree (data not shown).

### 18S and 28S rRNA genes

**Figure 3:**
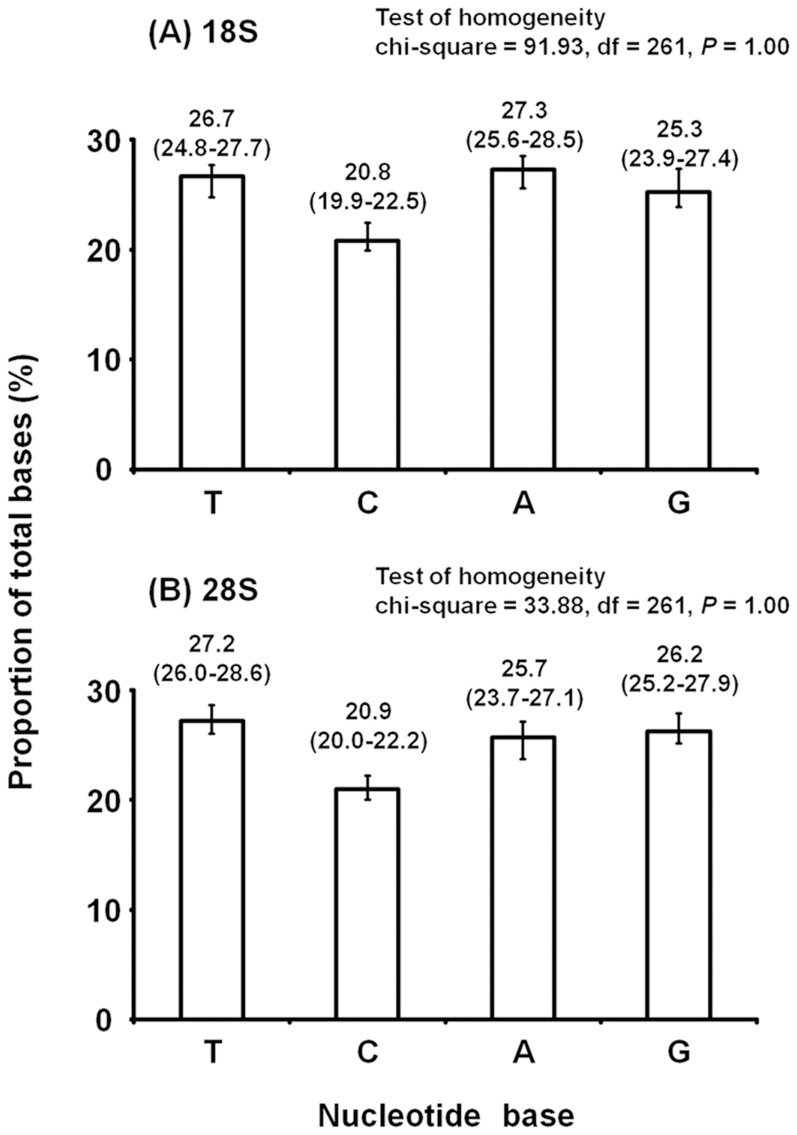
Base compositions of the (A) 18S and (B) 28S rRNA genes, averaged over all 88 mite strains used in this study. Error bars depict range. Results of the homogeneity test are given for each gene.

We determined the 18S and the 5′ end of the 28S rRNA sequences of all 88 strains used in this study (Table 1). The lengths of the 18S sequences obtained were 1,825–1,901 bp. The 18S and 28S sequences contained a number of gaps (insertions and deletions). After alignment and deletion of the ambiguous part of the aligned data, the final length was 1,863 bp, containing 495 parsimony-informative sites. The lengths of the 28S sequences were 646–743 bp, with a final length of 671 bp, containing 201 parsimony-informative sites. The aligned sequences before and after deleting the ambiguous parts are shown in Supporting Information (Files S2–S4). Chi-square tests revealed no significant heterogeneity in the nucleotide composition of the 18S and 28S sequences (Figure 3).

**Figure 4:**
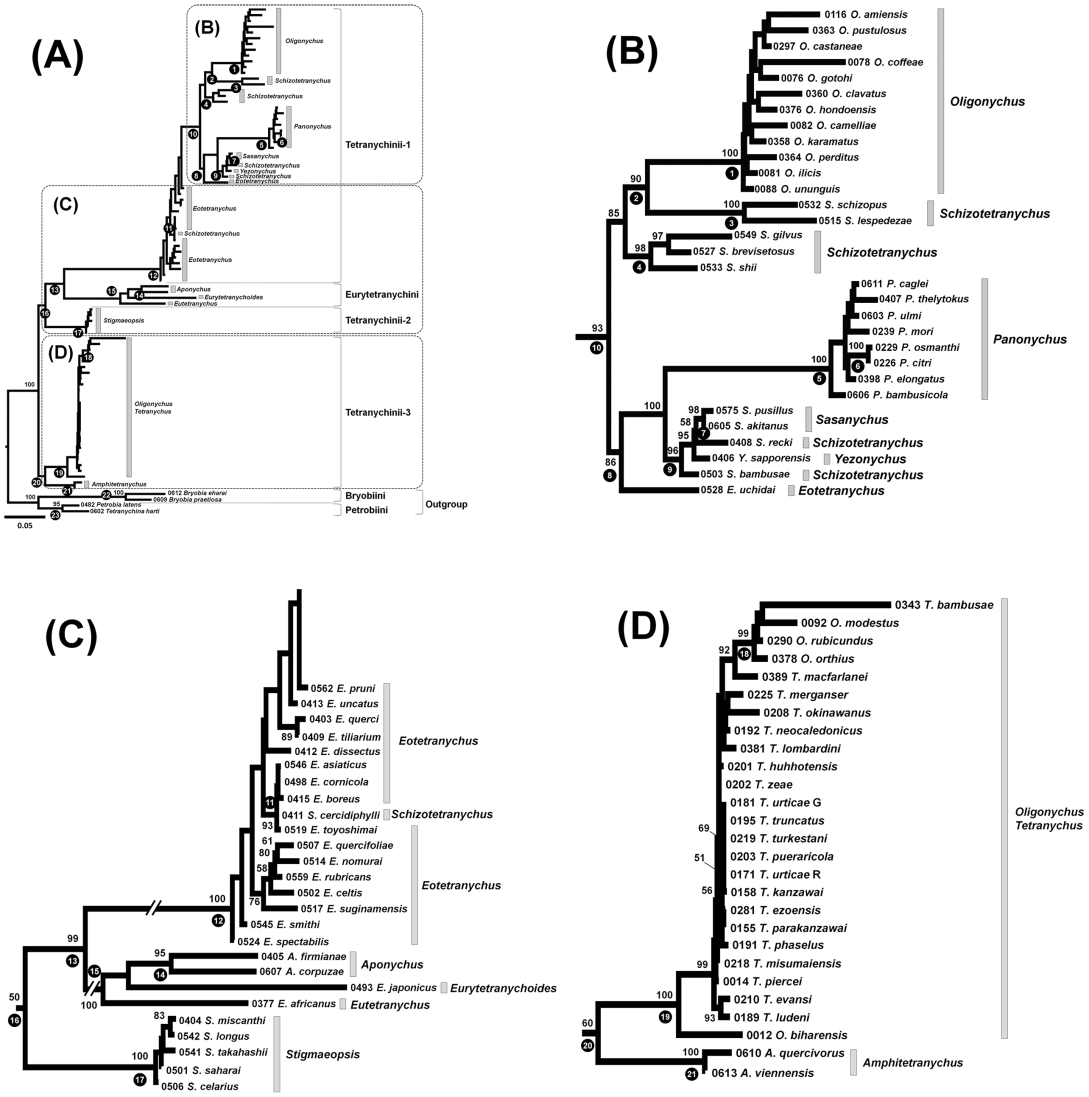
Maximum likelihood (ML) phylogenetic tree of the sub-family Tetranychinae based on the 18S and 28S rRNA genes using the GTR Gamma model. Bootstrap values (>50%) based on 1,000 replications are indicated at nodes. Each operational taxonomic unit is indicated by the voucher specimen no. and scientific name. Black circles with numbers indicate the clade no. which corresponds with the article. The tree is divided into three sections: (A) The entire tree, (B) Tetranychini-1, (C) Tetranychini-1, Eurytetranychini and Tetranychini-2 and (D) Tetranychini-3.

**Figure 5:**
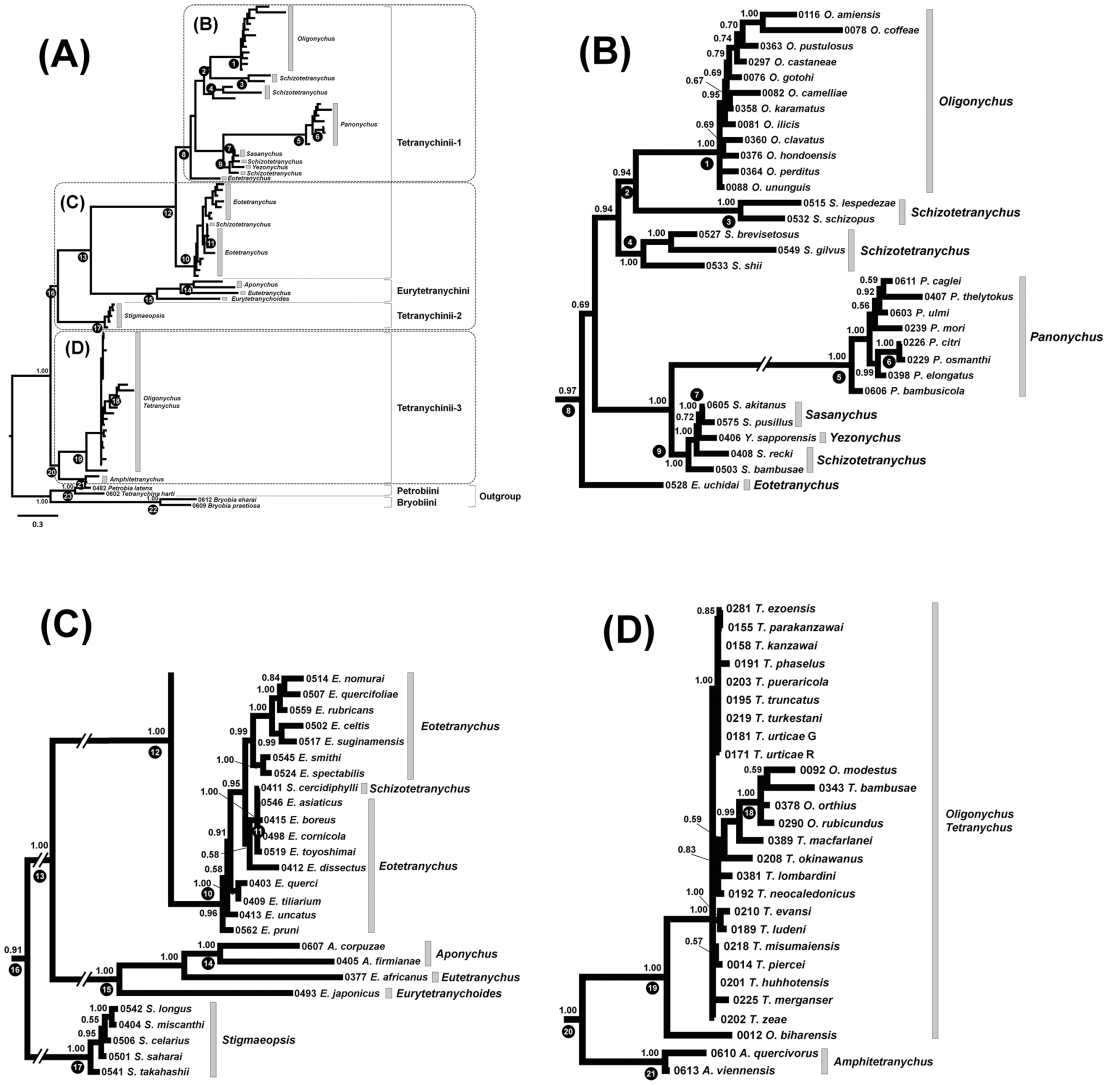
Bayesian phylogenetic tree of the sub-family Tetranychinae based on the 18S and 28S rRNA genes using the GTR Gamma model. Bayesian posterior probabilities (>0.50) are indicated at nodes. Each operational taxonomic unit is indicated by the voucher specimen no. and scientific name. Black circles with numbers indicate the clade no. which corresponds with the article. The tree is divided into three sections: (A) The entire tree, (B) Tetranychini-1, (C) Tetranychini-1, Eurytetranychini and Tetranychini-2 and (D) Tetranychini-3.

Phylogenetic trees based on a single gene were not as well resolved as phylogenetic trees based on the combined 18S and 28S data sets. Therefore, only the combined data set was used for the ML and Bayesian analyses. The 18S and 28S trees suggest that the tribes Bryobiini and Petrobiini of the sub-family Bryobiinae, which were used as outgroups, are both monophyletic (Figures 4A and 5A, clades 22 and 23). Within the Tetranychinae, Clade 15 is composed of species of Eurytetranychini, and clades 12,17 and 20 are composed of species of Tetranychini (Figures 4A and 5A). Among the 10 genera for which more than two strains were sampled, six genera (*Bryobia*, *Aponychus*, *Panonychus*, *Sasanychus*, *Stigmaeopsis* and *Amphitetranychus*), appear to be monophyletic with >95 bootstrap values and 1.00 posterior probabilities, while four genera (*Oligonychus*, *Tetranychus*, *Schizotetranychus* and *Eotetranychus*) are polyphyletic. The monophyletic genera are in clades 22, 14, 5, 7, 17 and 21, respectively (Figures 4A–4D and 5A–5D). Species of the genus *Oligonychus* are separated into 2 clades (clades 1 and 19), with the *Tetranychus* species included in clade 19 (Figures 4B, 4D, 5B and 5D). *Schizotetranychus* species, with the exception of *S*. *cercidiphylli*, are separated into 3 clades (clades 3, 4 and 9), with the *Sasanychus* and *Yezonychus* species included in clade 9 (Figures 4B and 5B). In the ML tree (Figures 4B–4C), *S*. *cercidiphylli* and *Eotetranychus* species, with the exception of *Eotetranychus uchidai* Ehara, are paraphyletic with respect to clade 10. *E*. *uchidai* forms a sister group with *Panonychus*, *Sasanychus*, *Schizotetranychus* and *Yezonychus* species (Figure 4B, clade 8). In the Bayesian tree (Figures 5B–5C), a well-supported clade consisting of *S*. *cercidiphylli* and *Eotetranychus* species, with the exception of *E*. *uchidai* (clade 10: Bayesian posterior probabilities (BPP) = 0.96) clustered with clade 8.

As was observed in the COI tree, the 18S and 28S trees also show the monophyly of *P*. *citri* and *P*. *osmanthi* which are closely related species (Figures 4B and 5B, clade 6). *S*. *cercidiphylli* forms a well-supported clade with four *Eotetranychus* species (*E*. *asiaticus*, *Eotetranychus boreus* Ehara, *E*. *cornicola* and *Eotetranychus toyoshimai* Ehara & Gotoh) in both ML and Bayesian trees (Figures 4C and 5C, clade 11: BP/BPP = 93/1.00). On the other hand, closely related *Eotetranychus* species (*Eotetranychus pruni* (Oudemans), *Eotetranychus querci* Reeves and *Eotetranychus uncatus* Garman), which have long, flagellate and undulate aedeagi [25], did not cluster together in either tree (Figures 4C and 5C).

## Discussion

Only a few studies have examined the molecular phylogeny of the sub-family Tetranychinae, and they often used genes or regions that had limited discriminating ability. As observed in previous studies, our tree based on the COI gene did not resolve deep-level phylogeny because of the low bootstrap values for deep nodes of tree (Figure 2). Therefore, we used the 18S and 28S rRNA genes for phylogenetic analyses because of their better discriminating ability. Indeed, our phylogenetic tree of the 18S and 28S sequences revealed several well-supported clades, allowing us to consider the phylogenetic relationships among the sub-family Tetranychinae.

Our phylogenetic trees based on the 18S and 28S rRNA genes suggest that the tribes Bryobiini and Petrobiini of the sub-family Bryobiinae are both monophyletic, but the tribe Tetranychini is polyphyletic because the monophyletic clade of Eurytetranychini is placed inside Tetranychini (Figures 4A and 5A). At the generic level, 4 genera (*Oligonychus*, *Tetranychus*, *Schizotetranychus* and *Eotetranychus*) are polyphyletic. The phylogenetic tree separates the *Oligonychus* species into two clades (Figures 4B, 4D, 5B and 5D, clades 1 and 19). That is, the two clades comprising the genus *Oligonychus* coincide with their morphology based on the direction of curvature of the aedeagus. These results are in agreement with our COI phylogeny (Figure 2) and previous phylogenies based on the COI gene and ITS2 region [10], [12], [13], [14]. Although phylogenies based on the COI gene and ITS2 region could not establish the exact phylogenetic positions of the two clades of *Oligonychus*, our tree suggests that species whose aedeagi curve ventrally form a sister group with some of the *Schizotetranychus* species (Figures 4B and 5B, clade 2) and species whose aedeagi curve dorsally are more closely related to *Tetranychus* species whose aedeagi also curve dorsally (Figures 4D and 5D, clade 19). Though *Oligonychus* and *Tetranychus* can be distinguished by their empodium shape, our phylogenetic trees reveal that the shape of the aedeagi can help to discriminate these two genera.

Species of the genus *Schizotetranychus* and *Eotetranychus* appear to be polyphyletic within clade 12 (Figures 4B–4C and 5B–5C). Puzzlingly, *S*. *cercidiphylli* and *E*. *uchidai* are separated from other congeneric species in the tree. The placement of *Eotetranychus* species is different between the ML and Bayesian trees. In the ML tree (Figures 4B–4C), we could not establish the exact phylogenetic position of the species of *Eotetranychus* which are paraphyletic respect to clade 10 because bootstrap values are relatively low. On the other hand, in the Bayesian tree (Figure 5C), *S*. *cercidiphylli* and the *Eotetranychus* species, with the exception of *E*. *uchidai*, clustered into a well-supported clade (clade 10: BPP = 0.96). Similarly, the phylogenetic position of the genus *Stigmaeopsis* is resolved in the Bayesian analysis but not in the ML analysis. In the ML tree (Figure 4C), *Stigmaeopsis* species (clade 17) clustered with clade 13, which includes the Eurytetranychini species and some of the Tetranychini species, but the topology is not well supported (clade 16: BP = 50). In the Bayesian tree (Figure 5C), *Stigmaeopsis* species (clade 17) clustered with clade 13 with high Bayesian posterior probabilities (clade 16: BPP = 0.91). Although our data suggests that the Bayesian tree (Figures 5A–5D) is better supported than the ML tree (Figures 4A–4D), it is common knowledge that posterior probabilities are generally higher than bootstrap values [26].

Phylogenetic trees can be used to assess associations between spider mites and their host plants [13]. In the ML and Bayesian trees (Figures 4D and 5D), *Oligonychus* and *Tetranychus* species inhabiting gramineous plants (*O*. *orthius*, *O*. *modestus*, *O*. *rubicundus* and *T*. *bambusae*) clustered separately from other species and formed a monophyletic clade (Figures 4D and 5D, clade 18). Clade 4 includes *Schizotetranychus brevisetosus* Ehara, *Schizotetranychus gilvus* Ehara & Ohashi and *Schizotetranychus shii* (Ehara) which inhabit fagaceous plants (Figures 4B and 5B). Clade 9 include species irrespective of genus, which inhabit bamboo plants, *Sasanychus akitanus* (Ehara), *Sasanychus pusillus* Ehara & Gotoh, *S*. *bambusae*, *S*. *recki* and *Yezonychus sapporoensis* Ehara (Figures 4B and 5B). All *Stigmaeopsis* species inhabiting gramineous plants are separated from other Tetranychini species and appear to be monophyletic (Figures 4C and 5C, clade 17). These results indicate that the phylogenetic relationships of some species of spider mites are closely linked with their host plant, as reported in other phytophagous arthropods [27], [28], [29].

We consider the phylogenies of the Tetranychinae based on the 18S and 28S rRNA genes to be a major improvement over previous phylogenies because they reveal several well-supported clades that were not distinguished by phylogenetic relationships based on the COI gene and ITS2 region. Our finding that the tribe Tetranychini and four genera (*Oligonychus*, *Tetranychus*, *Schizotetranychus* and *Eotetranychus*) are polyphyletic indicates that the diagnostic morphological characters of tribes and genera of Tetranychinae need to be reconsidered. Although we examined a large number of species in this study, most of them were collected in Japan. Analyzing a number of undescribed genera remaining throughout the world may help achieve a deeper understanding of the phylogenetic relationships among the family Tetranychinae. In addition, a large number of nuclear genes need to be examined to resolve poorly understood relationships in the ML tree (Figures 4A–4D), such as the phylogenetic positions of the genera *Eotetranychus* and *Stigmaeopsis*.

## Materials and Methods

### Mites

Eighty-four strains representing 12 genera and two tribes in Tetranychinae, were used in this study and four strains of the tribes Bryobiini and Petrobiini of the sub-family Bryobiinae (Acari: Tetranychidae) were used as outgroups (Table 1). Mite samples that could be reared in the laboratory were maintained on leaf discs of common bean leaves (*Phaseolus vulgaris* L.), mulberry leaves (*Morus bombycis* Koidz.) or the original host plants placed on a water-saturated polyurethane mat in a plastic dish (90 mm diameter, 20 mm depth) at 25°C under a 16L-8D photoperiod until analysis. Samples that could not be maintained in the laboratory and samples that were imported from abroad were preserved in 99.5% ethanol for molecular analyses and 70% ethanol for morphological identification. Specimens were mounted in Hoyer’s medium and identified under phase-contrast and differential interference-contrast microscopes. Voucher specimens are preserved at the Laboratory of Applied Entomology and Zoology, Faculty of Agriculture, Ibaraki University under the serial voucher specimen numbers (Table 1).

### DNA extraction, amplification, cloning and sequencing

Total DNA was extracted from the whole body of each female individual by using a Wizard Genomic DNA Purification Kit (Promega). Live female individuals for DNA samples and female individuals for voucher specimen were obtained from the same leaf discs. A few of the strains could not be maintained in the laboratory. For these strains, DNA samples were obtained from ethanol-preserved female individuals. The PCR primers are given in Table 2. The mitochondrial COI fragments were amplified using primer sets C1-J-1718 [30] and COI REVA [8] for species of 12 genera (*Bryobia*, *Petrobia*, *Eurytetranychoides*, *Aponychus*, *Panonychus*, *Sasanychus*, *Schizotetranychus*, *Yezonychus*, *Eotetranychus*, *Oligonychus*, *Amphitetranychus* and *Tetranychus*) and primer sets C1-J-1718-stig and COI REVA-stig for species of the genus *Stigmaeopsis*. COI sequences for *Oligonychus* and *Tetranychus* species were obtained from previously published data [10], [11]. PCR amplification was performed with the following profile: 3 min at 94°C, followed by 35 cycles of 1 min at 94°C, 1 min at 45°C for COI, 60°C for 28S and 65°C for 18S and 1.5 min at 72°C. An additional 10 min at 72°C was allowed for last strand elongation. The resultant DNA solutions were purified by using MinElute PCR Purification Kit (Qiagen) and sequenced directly. Sequencing was carried out using the sequencing primers (Table 2) with a BigDye Terminator Cycle Sequencing Kit v.3.1 (Applied Biosystems) and on an ABI 3130×l automated sequencer.

**Table 2:** Primers used in polymerase chain reaction amplification and sequencing of the mitochondrial COI gene and the 18S and 28S rRNA genes.

Primer name			Sequence	Application	References
COI					
	C1-J-1718	Forward primer	5′-GGAGGATTTGGAAATTGATTAGTTCC-3′	PCR amplification & sequencing	Simon et al. [30]
	COI REVA	Reverse primer	5′-GATAAAACGTAATGAAAATGAGCTAC-3′	PCR amplification & sequencing	Gotoh et al. [8]
	C1-J-1718-stig	Forward primer	5′-GGAGGTTTTGGTAATTGGTTAATCCC-3′	PCR amplification & sequencing	This study
	COI REVA-stig	Reverse primer	5′-GAAAGAACATAATGAAAATGAGCAAC-3′	PCR amplification & sequencing	This study
18S					
	18S-1F	Forward primer	5′-ACCGCGAATGGCTCATTAAATCAGTT-3′	PCR amplification & sequencing	This study
	18S-2F	Forward primer	5′-TGGCCTCTGAGCCGACGATGTAT-3′	Sequencing	This study
	18S-2R	Reverse primer	5′-ACCCCATAGGTTCGACTGAAATC-3′	Sequencing	This study
	18S-5R	Reverse primer	5′-TCCAATAGATCCTCGTTAAAGGAT-3′	Sequencing	This study
	18S-8R	Reverse primer	5′-TCTCGTTCGTTATCGGAATTAAC-3′	Sequencing	This study
	18S-9F	Forward primer	5′-AGCTTCCGGGAAACCAAAGTTT-3′	Sequencing	This study
	18S-9R	Reverse primer	5′-AGGGCATCACAGACCTGTTATT-3′	Sequencing	This study
	18S-10F	Forward primer	5′-AGTTGGTGGAGTGATTTGTCTGGT-3′	Sequencing	This study
	18S-10R	Reverse primer	5′-ACAAAGGGCAGGGACGTAATCAA-3′	PCR amplification & sequencing	This study
28S					
	28v-5′	Forward primer	5′-AAGGTAGCCAAATGCCTCATC-3′	PCR amplification & sequencing	Hillis and Dixon [31], Palumbi [32]
	28jj-3′	Reverse primer	5′-AGTAGGGTAAAACTAACCT-3′	PCR amplification & sequencing	Hillis and Dixon [31], Palumbi [32]

### Data analysis

All sequences obtained were deposited in DDBJ/EMBL/GenBank International Nucleotide Sequence Databases under the accession numbers AB981203 to AB981240, AB926227 to AB926314 and AB926318 to AB926405 (Table 1). Sequences were aligned using the 'auto' option of the MAFFT software [33]. Gaps (insertions and deletions) included in the 18S and 28S rRNA sequences were treated using the 'automated1' option of the trimAl software [34], which trimmed ambiguous sites by using a heuristic selection of the automatic method based on similarity statistics. The homogeneity of nucleotide composition was checked using chi-square tests implemented in PAUP* version 4.0b10 software [35].

Maximum likelihood (ML) and Bayesian phylogenetic trees were constructed with RAxML [36] and MrBayes5D [37], respectively. We used the tribes Bryobiini and Petrobiini of the sub-family Bryobiinae as outgroups to root the tree. For all analyses, we used the GTR Gamma model selected by the Akaike Information Criterion (AIC) using the program Kakusan4 [38]. The RAxML search was executed for the best-scoring ML tree in one single program run (the ‘-f a' option) instead of the default maximum parsimony-starting tree. Statistical support was evaluated with 1,000 rapid bootstrap inferences. The MrBayes5D analyses were implemented with two parallel runs of 10 million generations each and using one cold and two incrementally heated Markov chains and sampling every 100 steps. Tracer v.1.6 [39] was used to assess if the search had reached stationarity and to check whether the sample sizes for each parameter (ESS>100) were adequate. The first 10% of the trees were discarded as burn-in and the consensus tree with Bayesian posterior probabilities was constructed based on the trees sampled after the burn-in.

## Supporting Information

File S1 Aligned COI sequences in FASTA format.
(ZIP)

File S2 Aligned 18S sequences in FASTA format.
(ZIP)

File S3 Aligned 28S sequences in FASTA format.
(ZIP)

File S4 Aligned 18S sequences after deleting the ambiguous parts in FASTA format.
(ZIP)

File S5 Aligned 28S sequences after deleting the ambiguous parts in FASTA format.
(ZIP)
